# Cholangiocarcinoma in the Era of Immunotherapy

**DOI:** 10.3390/vaccines11061062

**Published:** 2023-06-05

**Authors:** Eleni Manthopoulou, Daryl Ramai, Jahnvi Dhar, Jayanta Samanta, Alexandros Ioannou, Ekaterina Lusina, Rodolfo Sacco, Antonio Facciorusso

**Affiliations:** 1 Department of Gastroenterology, St. Savvas Oncology Hospital of Athens, 11522 Athens, Greece; manthopoulouelena@gmail.com; 2 Gastroenterology and Hepatology, University of Utah Health, Salt Lake City, UT 801385, USA; daryl.ramai@hsc.utah.edu; 3 Department of Gastroenterology, Sohana Multi-Speciality Hospital, Mohali 140308, India; jahnvi3012@gmail.com; 4 Department of Gastroenterology, Post Graduate Institute of Medical Education and Research, Chandigarh 160012, India; dj_samanta@yahoo.co.in; 5 Department of Gastroenterology, Alexandra General Hospital, Lourou 4-2, 11528 Athens, Greece; alexandros.ioannou@gmail.com; 6 Therapeutic Unit, Gastroenterology Department, Chaika Clinics, Lesnaya Street 9, 125196 Moscow, Russia; eka-lusina@yandex.ru; 7 Department Medical and Surgical Sciences, University of Foggia, Viale Luigi Pinto 1, 71122 Foggia, Italy; antonio.facciorusso@virgilio.it

**Keywords:** cholangiocarcinoma, immunotherapy, adoptive cell therapy, cancer vaccines, checkpoint inhibitors

## Abstract

Cholangiocarcinoma (CCA) is a rare malignancy of the gastrointestinal tract, with aggressive behavior, and portends a poor prognosis. Traditionally, it is classified according to its site of involvement as intrahepatic, perihilar, and distal cholangiocarcinoma. A host of genetic and epigenetic factors have been involved in its pathogenesis. Chemotherapy has remained the standard first-line treatment over the last decade, with a disappointing median overall survival of 11 months for locally advanced and metastatic CCA. The advent of immunotherapy has revolutionized the treatment of many pancreaticobiliary malignancies, offering durable responses with a safe therapeutic profile. To date, there have been no significant advances in the management of CCA. Novel immunotherapeutic methods, such as cancer vaccines, adoptive cell therapy, and combinations of immune checkpoint inhibitors with other agents, are currently under investigation and may improve prognosis with overall survival. Efforts to find robust biomarkers for response to treatment along with multiple clinical trials are also ongoing in this regard. In this review, we present an overview of the current advances and the future perspectives of immunotherapy in the management of CCA.

## 1. Introduction

Cholangiocarcinoma (CCA) is part of an umbrella spectrum of “biliary tract cancers” (BTCs) and represents the most rare but aggressive of all biliary malignancies. CCA is characterized anatomically into intrahepatic CCA (iCCA), perihilar CCA (pCCA), and distal CCA [1]. It is second to hepatocellular carcinoma (HCC) in terms of prevalence, accounting for 15% of primary liver malignancy and 3% of all gastrointestinal cancers [2]. The incidence and mortality of CCA have been on the rise over the last 3 decades. It is more prevalent in elderly males, and several risk factors have been described, such as obesity, viral hepatitis with/without cirrhosis, primary sclerosing cholangitis (PSC), diabetes mellitus (DM), hepatolithiasis, and liver fluke infections. Despite this, noteworthily, around 50–60% have no identifiable causative factor [3,4].

The majority of cases present with non-specific symptoms leading to delayed diagnosis. Surgical resection is the only potential curative treatment option, but it is used in only 25% of cases, albeit with high morbidity and mortality. The vast majority present with disseminated/metastatic disease at diagnosis; hence, systemic chemotherapy (combination of gemcitabine and cisplatin) is currently the first-line treatment option for CCA, since 2010, based on the results of two landmark trials, the phase III ABC-02 and phase II BT22 trials [5,6,7]. Nevertheless, the overall survival (OS) rates are quite modest (11–12 months).

CCA being a chemo-refractory disease has subsequently fostered the parallel development of omics-based and novel targeted therapies for its management. More than half of CCAs harbor unique genetic aberrations such as fibroblast growth factor receptor (FGFR) 2 gene translocations, isocitrate dehydrogenase-1 (IDH1) and KRAS proto-oncogene mutations, and receptor tyrosine-protein kinase erbB-2 (ERBB2) amplification [8]. Currently, Pemigatinib, Infigratinib, Ivosidenib, Zanidatamab, and Futibatinib have been granted Food and Drug Administration (FDA) approval or breakthrough therapy designation for CCA management [9,10]. Despite this, none of the targeted therapies have been approved as first-line treatment options for CCA.

Emerging advances towards a personalized treatment plan for CCA with a paucity of efficacious therapeutic options have opened up new horizons for such a highly challenging biologically heterogeneous malignancy. On these lines, immuno-oncology has revolutionized the therapeutic arsenal for various solid organ malignancies such as kidney, lung, and colon cancers with promising results, but its use in BTCs merits further investigation. Currently, it stands as a fifth pillar of cancer treatment for oncologists, namely after surgery, chemotherapy, radiation, and omics-based targeted approach. The term “immunotherapy” entails three options: immune checkpoint inhibitors (ICIs), cancer vaccines, and adoptive cell transfer. The former includes programmed death 1 (PD-1), programmed death-ligand 1 (PD-L1), and cytotoxic T-lymphocyte antigen-4 (CTLA-4) [1,2].

The primary mechanism of immunotherapy aims to enhance natural antitumor responses of the infiltration of both innate cells (neutrophils, macrophages, dendritic cells, natural killer cells) and adaptive cells (T and B lymphocytes) into the tumor microenvironment (TME) as well as inhibition of immune checkpoints, which results in the restoration or improvement of anticancer immunity and a reduction in cancer volume [1,2]. Immune checkpoints regulate the immune response to invading foreign molecules under normal conditions. Tumor cells interact with immune checkpoints, thereby inhibiting immune surveillance. The immune checkpoints are typically PD-1 and CTLA-4. T-cell apoptosis is then induced by the activation of checkpoints by their specific surrogates (PD-L1 for PD-1 and CD152 for CLT-4). PD-1 binding to PDL-1 and PDL-2 inhibits T-cell activity and promotes tumor development. As T-cell immune activity is restored, blocking PD-1 may enhance antitumor activity by restoring T-cell immune activity [1,11].

CCA is characterized by a prominent desmoplastic stroma, consisting primarily of cancer-associated fibroblasts (CAFs) and a highly reactive TME. This immune landscape within the TME of CCA holds tremendous potential in terms of identifying responsiveness to immunotherapy with subsequent novel treatment strategies. The ICI-based therapy is conceptualized on the above aspect, as those who have hot and altered immunosuppressed phenotypes benefit greatly with gratifying results, while those with altered–excluded and cold fare poorly to immunotherapy [12]. The TOPAZ-1 trial marks the first example in this regard highlighting the therapeutic success in advanced BTCs [13].

**Figure 1:**
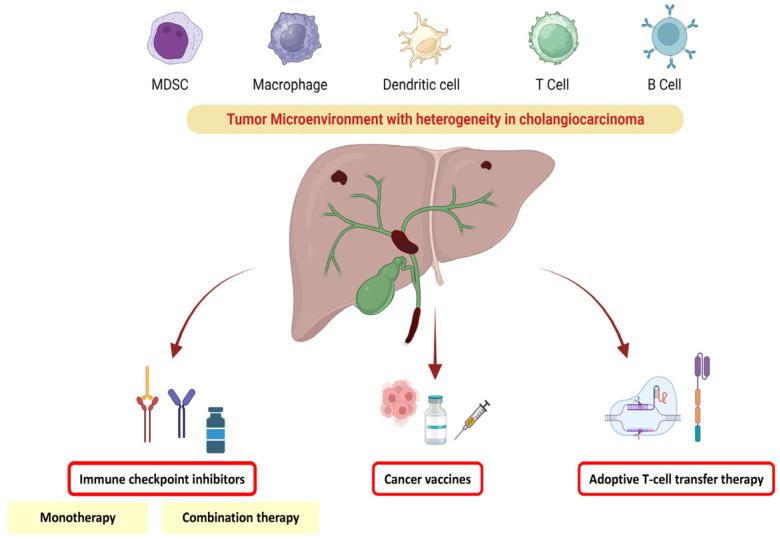
Potential therapeutical targets for immunotherapy in cholangiocarcinoma.

Hence, this review will help elucidate the concept of immunotherapy in cholangiocarcinoma and understand the concept of tumor heterogeneity and potential future therapeutic targets, covering from the incipient stage to current ongoing trials (Figure 1).

## 2. Literature Search

A detailed strategy, using the keywords “cholangiocarcinoma” AND “immunotherapy” was performed in PubMed. All studies pertaining to the role of immunotherapy in CCA as well as biliary tract cancers were reviewed (case series, review articles, and clinical trials). A brief discussion on the concept of heterogeneity in CCA is presented in this review. Non-English language literature was excluded. Topics concentrating on targeted therapies in CCA/BTC are beyond the scope of this review and have been excluded.

## 3. Tumoral Heterogeneity in Cholangiocarcinoma and Impact on Therapeutic Management

CCA tumor biology is defined with a complex enriched TME, and the presence of cells of innate immunity (neutrophils CD163+ M2 macrophages) has been inversely associated with poor outcomes in its management. CCA can be divided into mass-forming, intraductal, and periductal infiltrating subtypes. All three are extremely heterogenous and exhibit high plasticity. This occurrence of heterogeneity at the molecular and cellular levels facilitates stratification based on immune profiles and guides decision making [14,15].

Intertumoral and intratumoral heterogeneity is a well-known phenomenon in CCA. In 566 CCA cases, Job et al. reported four TME molecular subtypes: (1) immune desert, (2) immune activation, (3) myeloid-rich, and (4) mesenchymal-like. Out of these subtypes, immune activation CCA was associated with the strongest ICI response, and mesenchymal-like with was associated with the poorest survival [16].

Similarly, with the development of single-cell technology, intratumoral CCA dynamics have been studied thoroughly. Within the same tumor, the T-cell repertoire (CD4, CD8) exhibits different subtypes of PD-1, CTLA-4, TIGIT, and more. Kim et al. showed prolonged PFS (HR 0.23) in those who received nivolumab in advanced BTC with PDL-1 expression (median PFS 3.68 months and median OS 14.24 months) [17]. On the contrary, Monge et al. did not reveal this association with pembrolizumab with oxaliplatin–capecitabine [18]. These contradictory results highlight potential unsolved roles of T cells, exosomal vesicles, CCA TME, and genetics. Hence, a detailed picture of the complete molecular, cellular, and TME profile is needed in elucidating the responses to immunotherapy in CCA.

## 4. ICI Immunotherapy

### 4.1. Keynote 28

Keynote 28 was a phase I study that evaluated the efficacy of the anti-PD-1 monoclonal antibody pembrolizumab in treating patients with various types of solid tumors (10 mg/kg every 2 weeks for 2 years). This study included 24 patients with histologically or cytologically confirmed CCA who had progressed despite receiving standard treatment. The primary inclusion criterion for this study was PD-L1 positivity (greater than 1% tumor and tumor-associated cell expression) with a median follow-up of 5.7 months. Pembrolizumab demonstrated durable antitumor activity in 13% of patients with advanced CCA. The objective response rate (ORR) was 13.0%. The median overall survival (OS) was 5.7 months, with a median progression-free survival (PFS) of 1.8 months (Table 1). Only one patient prematurely discontinued treatment due to autoimmune hemolytic anemia. Around 16.7% of them experienced grade 3 adverse events (AEs). The most common AEs were fever (16.7%), nausea (12.5%), and pruritus (12.5%). There were no grade 4 or 5 immune-mediated AEs or infusion reactions [19].

### 4.2. Keynote 158

Thereafter, a phase II study wherein PD-L1-positive tumors were not required for patient eligibility was conducted. Keynote 158 included 104 patients with recurrent and advanced CCA who received intravenous infusions of pembrolizumab (200 mg every 3 weeks for 2 years or until disease progression or intolerable toxicity). The median duration of follow-up was 7.5 months. ORR was 5.8%, and OS was 7.4 months, with a PFS of 2.0 months. On further subgroup analysis, those with PD-L1-positive tumors had an ORR of 6.6%, a median PFS of 1.9 months, and a median OS of 7.2 months, whereas those with PD-1-negative tumors had an ORR of 2.9%. In this phase II study, 12.5% of patients experienced grade 3 adverse events, and 5.8% discontinued treatment due to adverse effects, which included pneumonitis, renal failure, elevated liver enzymes, and hepatocellular damage. The most common AEs were fatigue (14.4%), rash (11.5%), and pruritus (8.2%). Hence, it was concluded that pembrolizumab monotherapy provided durable antitumor activity independent of PD-L1 expression with manageable toxicity in patients with advanced CCA [19].

### 4.3. Nivolumab

Nivolumab is a human immunoglobulin G4 anti-PD-1 agent and was evaluated with and without chemotherapy (cisplatin and gemcitabine) in a Japanese cohort phase 1 study. This study included two groups, each including 30 patients with unresectable or recurrent biliary tract cancers (BTCs) that were refractory/intolerant to gemcitabine-based treatment: one received monotherapy with 240 mg nivolumab every 2 weeks, and the second group was allocated to same dose of nivolumab with cisplatin (25 mg/m^2^)–gemcitabine (1000 mg/m^2^) combination. While the monotherapy cohort had a median OS of 5.2 months and a median PFS of 1.4 months, with only 1 of 30 patients showing an objective response, the combination therapy cohort demonstrated a median OS of 15.4 months and a median PFS of 4.2 months, and 11 of 30 patients had an objective response [20,21].

Nivolumab was further evaluated in a multicenter phase II study involving 54 patients with histologically confirmed CCA whose disease progressed while being treated with at least one line but no more than three lines of systemic chemotherapy. It was delivered intravenously (IV) (240 mg) every 2 weeks for 16 weeks, and then 480 mg was delivered IV every 4 weeks until disease progression or the presence of unacceptable AEs. The ORR achieved was 22%, and the disease control rate was 59%. All patients who responded to treatment were not MSI-H. The median PFS was 3.68 months and the median OS was 14.24 months in the intention-to-treat population. PDL1-1 expression in tumors was associated with prolonged progression-free survival. The most common adverse events were hyponatremia (6%) and elevated alkaline phosphatase (4%) [17].

These clinical studies confirmed the clinical efficacy of nivolumab for advanced CCA, either as monotherapy or in combination, with an acceptable AE profile.

### 4.4. Durvalumab

In a phase I study, durvalumab (anti-PD-L1) with or without tremelimumab (anti-CTLA-4) was evaluated in advanced solid tumors, including CCA. Two out of forty-two patients who received durvalumab as monotherapy had a partial response (ORR = 4.8), compared to 7 of 65 patients who received the combination therapy (ORR = 10.8). The median OS was 8.1 months for monotherapy versus 10.1 months for the combination group. Around 64% of patients in the monotherapy group versus 82% receiving combination therapy experienced treatment-related AEs of any grade. Two patients in the monotherapy cohort and five in the combination therapy cohort discontinued treatment as a result of adverse events [22].

### 4.5. Bintrafusp Alfa

Bintrafusp alfa inhibits the proliferation of malignant cells by combining the mechanistic action of two proteins, transforming growth factor (TGF-beta) and PD-L1, making it a bifunctional fusion protein. This proof of concept was shown in the first phase I study wherein 20 Asian patients (diagnosed with metastatic/locally advanced BTC) who had failed first-line treatment received bintrafusp alfa (1200 mg every 2 weeks). Six patients achieved OR (20%), while only two had a complete response (CR). The median PFS was 2.5 months, and the median OS was 12.7 months. The most common AEs were rash (17%), fever (13%), papular rash (13% each), and increased lipase (10%) [23].

Following this, a phase II study wherein this drug was given as second-line treatment in 159 cases of advanced/metastatic BTC was conducted. Although the ORR was 10.1%, the predefined threshold allowing its use as second-line therapy was not reached [23,24,25].

## 5. Combination Therapies

Due to the overall limited activity of ICI monotherapy in metastatic BTC (up to 20% ORR) [19,20,21,22,23], a number of studies on the efficacy of combination therapies have been conducted to assess their response in the management of CCA [26,27]. These involved the use of two distinct immunotherapy agents: immunotherapy in conjunction with chemotherapy or immunotherapy in conjunction with antiangiogenic agents.

### 5.1. ICI Combination Therapy

Cytotoxic T-lymphocyte-associated antigen-4 (CTLA-4) modulates the early immune response, whereas PD-L1 modulates the late immune response in peripheral tissue [11]. Combining CTLA-4 and PD-1 inhibitors could have a synergistic antitumor effect, resulting in enhanced activity and increased infiltration of tumor-infiltrating lymphocytes (TILs) as well as a decrease in regulatory T cells (Tregs) in the CCA microenvironment [26,27] (Table 2).

In a multicenter phase II study involving 39 cases of metastatic BTC (16 intrahepatic CCA, 10 extrahepatic CCA, 13 gall bladder cancer), the combination of nivolumab (anti-PD-1 inhibitor) and ipilimumab (anti-CTLA-4 inhibitor) was investigated. Thirty-three patients (85%) had previously received a minimum of one line of systemic treatment. Nivolumab (3 mg/kg) and ipilimumab (1 mg/kg) were administered every 3 weeks for four cycles, followed by nivolumab every 2 weeks for up to 96 weeks or until disease progression or unacceptable toxic events. An ORR of 23% was observed in 9 of 39 patients, with a disease control rate (DCR) of 44%. The median PFS was 2.9 months, with a median OS of 5.7 months. Immune-related toxic events were reported in 49% of patients, with 15% experiencing grade 3–4 events [28]. Hence, combination therapy with nivolumab and ipilimumab showed a substantial response in advanced intrahepatic CCA cases, warranting future trials in this regard.

On similar lines, the efficacy of the nivolumab and ipilimumab combination was also investigated in a multi-institutional phase 2 clinical trial for patients with advanced BTC who had not previously received systemic therapy. The first group of patients received gemcitabine, cisplatin, and nivolumab, while the other received nivolumab and ipilimumab. Treatment was administered to 68 of the 75 randomized patients (arm A = 35, arm B = 33). The PFS at 6 months was 59.4% in arm A and 21.2% in arm B. The median PFS and OS were 6.6 and 10.6 months in arm A and 3.9 and 8.2 months in arm B, respectively. Neutropenia (34.3% in arm A) was the most common treatment-related grade 3 or higher hematologic adverse event, with fatigue (8.6% in arm A) and elevated transaminases (9.1% in arm B) being the most common non-hematological side effects [29]. Thus, in this trial, combination therapy did not improve the 6-month PFS rate.

As previously mentioned, the efficacy and safety of a similar dual ICI regimen (durvalumab and tremelimumab) were assessed in a phase 1 trial on 65 Asian patients with advanced BTC. Every four weeks, the patients received 20 mg/kg of durvalumab and 1 mg/kg of tremelimumab. Seven patients (11%) experienced an ORR of 10.8%, with a median OS of 10.1 months, a median PFS of 1.6 months, and a median duration of response (DOR) of 8.5 months. The disease control rate was 32.2% in 12 weeks. Grade 3 treatment-related AEs occurred in 23% of patients. Five patients discontinued the therapy, and one death was reported due to drug-induced liver injury [22,30].

### 5.2. ICIs and Chemotherapy

Chemotherapy’s antitumor effect has been linked not only to its cytotoxic effect on tumor cells but also to the potential induction of the host immune response. This may enhance cancer cell immunogenicity by inducing immunogenic cell death (ICD). Dying cells release neoantigens and immunostimulatory molecules such as damage-associated molecular patterns (DAMPs) and cytokines [1,2,31]. Another antitumor mechanism of chemotherapeutic agents is the inhibition of immune evasion mechanisms used by tumors. The majority of chemoimmunotherapy trials in advanced BTC used gemcitabine–cisplatin (GEMCIS) as the primary chemotherapy base, though oxaliplatin-based regimens have also been used [2] (Table 3). The combination of chemotherapy and ICIs is a standard of care for a wide range of cancers, including NSCLC, gastric cancer, esophageal cancer, urothelial carcinoma, and breast cancer [31].

As highlighted previously, Ueno et al. conducted a trial to study the efficacy of immunotherapy in CCA [20]. In this trial, patients with unresectable or recurrent CCA who were gemcitabine-intolerant received monotherapy with nivolumab (n = 30), and those who were chemotherapy-naïve received the combination therapy of nivolumab with GEMCIS (n = 30). The former treatment group had only one patient who achieved an objective response, with a median OS of 5.2 months and a median PFS of 1.4 months. On the other hand, the combination therapy arm showed 11 of 30 patients with an objective response, with a median OS of 15.4 months and a median PFS of 4.2 months [20].

Feng et al. tested the GEMCIS and nivolumab combination in 32 patients with metastatic or incurable BTC. The combination therapy group demonstrated a median OS of 6.1 months and an average survival of 8.5 months. Among those who had previously received chemotherapy and those who did not, there were no considerable differences in median PFS and OS. Furthermore, PD-L1 expression was not related to survival or overall responses [32]. In both studies, the most common side effects of combination therapy were leukopenia and thrombocytopenia, with a manageable overall adverse event profile [20,32].

The efficacy and safety of camrelizumab, gemcitabine, and oxaliplatin (GEMOX) as first-line therapy were assessed in the phase II trial by Chen et al. The median PFS rate was 50%, and the median PFS and OS were 6.1 and 11.8 months, respectively. Fatigue (73%) and fever (73%) were the two most common treatment-related AEs. The ORR was 80% in those with a PD-1-positive tumor proportion score of ≥1% [34]. Similarly, in a phase II study by Chen et al. with advanced CCA cases, camrelizumab was also investigated in combination with chemotherapy. The two treatment arms were as follows: group 1 (n = 29) received camrelizumab plus 5-fluorouracil, leucovorin, and oxaliplatin (FOLFOX4), and group 2 (n = 63) received camrelizumab plus gemcitabine and oxaliplatin (GEMOX). The median PFS and OS were 5.3 months and 12.4 months, respectively. Grade ≥3 AEs occurred in 82.8% of the Cam-FOLFOX4 group and 68.3% of the Cam-GEMOX group [34].

A retrospective Chinese study compared the combination of chemotherapy plus PD-1 inhibitors (75 cases) with a chemotherapy-alone group (59 cases). The median PFS was 5.8 vs. 3.2 months, respectively, with no substantial difference in tumor response (assessed using ORR and DCR). DCR was 80.4% and ORR was 21.7% in the combination group. Hepatitis, rash, and hypothyroidism were the grade 3–4 treatment-related adverse events (AEs) in the anti-PD-1 + C group, but the rates of leukopenia (grade 3–4) were similar between the two groups (4.3% vs. 6.5%) [35].

As a first-line treatment for advanced BTC, the TOPAZ-1 trial (phase III trial) showed that the combination of durvalumab–GEMCIS therapy significantly improved survival (median 12.8 vs. 11.6 months, respectively) when compared to the CT-alone group. Detailing this RCT further, patients were divided into two treatment arms: one receiving durvalumab with GEMCIS for eight cycles followed by durvalumab for four cycles and another receiving placebo until disease progression or unacceptable toxicity. The median OS for the combination vs. placebo arm was 12.9 vs. 11.3 months, respectively. The OS rates at 1 and 2 years were 54.3% vs. 47.1% and 23.6% vs. 11.5%, respectively. Furthermore, adding durvalumab to gemcitabine and cisplatin improved PFS (7.2 months vs. 5.7 months) and ORR (26.7% vs. 18.7%) [36]. Grade 3 or higher adverse events were comparable between the two groups (60.9% vs. 63.5%), possibly indicating that chemotherapy alone is responsible for the adverse event profile. One intriguing finding in the study was that patients were provided with a CT-free period and a remarkable 2-year OS rate. Moreover, this study also exhibited a higher survival rate in the Asian population as compared to the remaining population [36].

The racial disparity in the response to ICIs has been shown to be true even for other malignancies (such as NSCLC, head and neck cancers, breast cancer, and esophageal and gastric cancers), wherein ICI therapy has shown higher clinical responses (survival outcomes) in Asian populations. A recent systematic review and meta-analysis has reinforced the above results, showing higher clinical responses (OS and PFS) to PD-1 and PD-L1 therapy in Asians. The overall estimated hazard ratio (HR) for Asian vs. non-Asian cancer patients was 0.69 vs. 0.82 for OS and 0.54 vs. 0.69 for PFS, respectively [37]. Few explanations have been postulated for this racial response to immunotherapy across varied malignancies including CCA: Firstly, it has been proposed that ethnic differences in somatic mutations (such as *STK11, TP53,* and *EGFR*) existing between Asians and non-Asian populations, referred to as “Asian signature”, affect the efficacy of ICIs [38]. Secondly, the clearance of antibodies affects the clinical response to ICIs between Asian and Caucasian ethnicities [39]. Hence, all these factors must be taken into due consideration while assessing OS/PFS for ICIs in a varied ethnic cancer population.

Furthermore, another phase II study evaluated the combination of durvalumab, tremelimumab, and chemotherapy in 121 chemotherapy-naïve metastatic BTC tumors. The biomarker cohort (BMC) included 33 patients who received GEMCIS for one cycle and then received GEMCIS, durvalumab, and tremelimumab every 3 weeks until disease progression. In the second group, 45 patients were treated with GEMCIS and durvalumab in three combined cohorts. The third group had 46 cases who received GEMCIS, tremelimumab, and durvalumab. The ORR was equivalent in second and third groups and lowest for the BMC group (73.3% vs. 73.4% vs. 50%) with a median PFS of 11.9 vs. 11 vs. 13 months, respectively. The median OS was 15 months, 18.1 months, and 20.7 months, respectively [2,40].

### 5.3. ICIs and Antiangiogenic Agents

BTC is characterized by increased expression of neo-angiogenic pathways, including VEGF [26]. The addition of an antiangiogenic agent may improve immunotherapy activity by contributing to lymphocyte cytotoxicity in the tumor microenvironment [2] (Table 4).

Lenvatinib is an antiangiogenic multi-kinase inhibitor. The efficacy and safety of its combination with pembrolizumab were studied in 31 patients with advanced BTC treatment in the LEAP-005 trial. The ORR was 10%, the median PFS was 6.1 months, and the median OS was 8.6 months. Hypertension, dysphonia, and diarrhea were the most common AEs [41]. Similar results were obtained when regorafenib was combined with anti-PD-L1 avelumab [2].

Toripalimab and lenvatinib in combination with GEMOX were studied in a phase II study as first-line therapies for advanced iCCA. The ORR was 80% and the PFS was 10 months, but the OS was not achieved. Jaundice, rash, and proteinuria were common treatment-related AEs that were encountered [42].

## 6. Adoptive Cell Therapy

Adoptive cell therapy consists of the genetic modification of the T cells to express “chimeric” antigen receptors (CARs) or tumor antigen-specific T-cell receptors (TCRs), intensifying their ability to recognize and eliminate of malignant cells [43].

### 6.1. Chimeric Antigen Receptor T-Cell (CAR-T Cell)

CAR-T-cell therapy targets specific cancer-related antigens. A potential target for CCA is the receptor mucin 1 (MUC1) which is vigorously expressed and is associated with poor prognosis and short survival. Recently, a fourth-generation CAR-T cell containing anti-MUC1 domains has been created. Supimon et al. revealed cytotoxic effects on malignant cells, suggesting MUC1 as a potential therapeutic target for CCA [44]. An additional target, namely integrin ανβ6, is suggested to be upregulated in CCA. In vitro studies revealed high-level cytotoxicity in malignant cells in comparison with peripheric epithelial tissues in which there is no upregulation of integrin ανβ6 [45]. The epidermal growth factor HER-2 is highly expressed in 3–19% of the patients affected by biliary tract malignancies. In a phase I clinical trial conducted by Feng et al., 11 patients with advanced HER2-positive CCA were treated with cyclophosphamide and paclitaxel followed by HER2-targeted CAR-T cells with moderate results (one patient with partial response and five patients with stable disease) [46]. Another target in a phase 1 trial was the epidermal growth factor receptor (EGFR), which is also expressed in BTC. Guo et al. studied the response of 19 patients, 14 with CCA, who were previously treated with cyclophosphamide and paclitaxel and then received EGFR-targeted CAR-T therapy. Unfortunately, only 1 patient achieved a complete response while 10 had stable disease in the study period of 22 months [47]. Currently, investigative targets for BTCs are mesothelin, CD133, claudin 18.2, and prostate stem cell antigen [48]. A few studies have also suggested that an improvement of CAR-T cells could be achieved with an addition of PD-1/PD-L1 blockade in solid tumors [49,50].

### 6.2. Tumor-Infiltrating Lymphocytes (TILs)

TILs are immune cells that have been at the center of research for the treatment of BTC. The CCA tumor microenvironment leads to immunosuppression which promotes the replication of malignant cells [51,52]. CCA malignancies can be divided into those that present with immune cell infiltration and that do not, with better response to therapy in the former [53]. To date, the potential involvement of TILs in the immunotherapy of CCA is limited in animal and in vitro studies. In cell culture experimental studies, the death of malignant cells was increased with enhanced cytotoxicity by the combination of cetuximab with cytokine-activated killer cells [54,55]. Diggs et al. studied a combination therapy of anti-CD4/PD-1 agents in murine models of intrahepatic CCA and demonstrated highly activated natural killer (NK) cells and CD4+/CD8+ T cells, which impaired malignant cell growth and prolonged survival [56].

## 7. Vaccine Therapy

Cancer vaccines represent a rapidly evolving armamentarium in the field of cancer treatment research. Activation of the immune system of the patient, enhancing immunogenicity and inducing cellular and humoral immune responses to block neoplastic evolution, seems to be a promising therapeutic strategy [2]. Experimental studies on rats suggest the efficacy of a DNA vaccine targeting CTLA-4 and PD-1, while other studies focus on developing mRNA vaccines for CCA targeting tumor antigens (CD247, TRRAP, FCGR1A) [57,58]. Currently, there are two malignancy-specific antigens involved in cancer vaccine therapy: Wilms’ tumor gene antigen (WT1) and mucin 1 (MUC1). Both of them are highly expressed in BTCs and are associated with a worse prognosis [59]. To date, single-antigen vaccines are usually studied in clinical trials, while multi-antigen and dendritic cell vaccines mainly represent the future research direction in the therapeutic field of cancer vaccines [1]. A clinical trial studying the combination of gemcitabine and WT1 (single-antigen vaccine) involving eight patients with CCA revealed the safety and efficacy of the treatment as a combination therapy with comparable adverse events [60]. Similarly, multi-antigen vaccines seem to have better outcomes as a result of the recognition by MHC-I and MHC-II at the same time and the induction of CD4+/CD8+ cell response. Aruga et al. demonstrated a median OS of 9.7 months and a targeted immune response in all nine patients with advanced BTC using a multi-antigen vaccine (HLA-A2402 restricted epitope peptides—cell division associated 1 (CDCA1), cadherin 3 (CDH3) and kinesin family member 20A (KIF20A)) [61].

Finally, dendritic cell (DC) vaccines are future targets of research in the field of oncology. DCs represent powerful and effective antigen-presenting cells inducing the production of cytotoxic T cells and specific anti-neoplastic cell immunity [62]. A retrospective study of a group of patients with unresectable, metastatic CCA receiving DC-based immunotherapy revealed that 15% of patients had stable disease along with an acceptable AE profile [63]. Recently, a study demonstrated that a DC vaccine in combination with chemotherapy prolonged the overall survival time, in comparison with vaccine-only treatment. Jiraviriyakul et al. revealed that DC immunotherapy antitumor activity could be increased with cell lysates derived from a Honokiol-treated CCA cell line (KKU-213L5) [64].

## 8. Future Directions

The results of the TOPAZ-1 trial have opened new horizons in the management of chemo-refractory advanced biliary tract cancers. The addition of durvalumab to the GEMCIS regimen has been proposed to be the first-line therapy in BTCs, showing improved OS rates [36]. However, there are multiple factors that have been shown to affect the clinical efficacy of ICIs across various cancer treatment regimens. The main obstacles to this are the presence of inter/intratumoral heterogeneity and the lack of predictive biomarkers. The most important in this regard is the deficiency in mismatch repair (MMR) proteins and their phenotype (presence of microsatellite instability (MSI)). Solid tumors harboring MMR deficiency have been shown to exhibit markedly higher responses to ICIs [65]. However, only 3% of CCAs have MMR deficiency and MSI-H phenotype. A recent study evaluated the response to pembrolizumab in solid tumors harboring MMR deficiency/MSI-H phenotype. Therein, for CCA, OR was achieved in 41% of cases in a chemo-refractory population with a median OS of 23.4 months, making it an attractive option for future clinical trials [66].

In addition, ICIs have been evaluated with other agents as combination therapies, such as targeted therapy, antiangiogenic agents, local ablation, radioembolization, and intratumoral injection of chemotherapeutic agents. These approaches have been hypothesized to increase tumor antigen exposure or inhibit immunosuppressive elements such as Tregs, which increases immune response when combined with ICI therapy. This represents a fascinating area of future research.

Locoregional therapies are recently emerging therapies that have been tested in combination with ICIs. A study by Xie et al. combined tremelimumab (anti-CTLA-4) with microwave ablation in 20 treatment-refractory BTCs. Stable disease was achieved in 31.3%, and median OS and PFS were 6 and 3.4 months, respectively. This combination therapy has been hypothesized to increase circulating activated CD8^+^ T cells and expand the TCR repertoire, contributing to treatment benefit [67].

Novel immunotherapeutic options have been developed in the last 4 years and are undergoing investigational trials to study their impact on BTCs. Four different approaches have been selected: firstly, modulating the tumor microenvironment (cytokine modulation, extracellular matrix); second, manipulating the fecal microbiome (use of fecal microbiota transplantation, vancomycin); third, targeting immunogenic tumor peptides (TLR); and lastly, cellular therapies (HER2, NK cells). All these future directions can help in the clinical efficacy of immunotherapy for CCA or BTCs [68,69].

Another challenging and fascinating area of research for immunotherapy in cholangiocarcinoma is its effect on patients with PSC, autoimmune hepatitis, and other autoimmune diseases, as these individuals were excluded from trials such as TOPAZ. Patients such as these are frequently treated with corticosteroids and chemotherapy, and their management is complicated. The current ongoing trials on immunotherapy as a first-line treatment for advanced BTCs are tabulated in Table 5.

## 9. Conclusions

Immunotherapy, consisting of ICIs, tumor vaccines, and chimeric antigen T-cell receptor conjugates, has revolutionized the management of advanced/metastatic CCA. The role of immunotherapy is currently under investigation, but promising results in solid malignancies have accelerated its establishment in managing advanced CCA with improved patient outcomes. The spatial heterogeneity of CCA hinders the development of reliable biomarkers to help predict responsiveness to ICIs. The rapidly transforming landscape of immunotherapy for CCA faces several challenges. Firstly, the presence of a rich TME with immune evasion needs to be elucidated with a deeper understanding, as it regulates immune responses and influences the tumor efficacy of the ICIs (monotherapy or combination with CT). Secondly, the ever-growing armamentarium of combination therapy has shown excellent results in managing BTCs. The approach of combining ICIs with chemotherapy and/or targeted therapy has garnered clinical interest, and several trials are being undertaken in this regard, which is important from an immunotherapy perspective. The results of the TOPAZ-1 trial are the beginning of a new era prolonging the survival of CCA patients. Lastly, to date, no predictive biomarker (MSI/MMR status, PDL-1) exists or has been validated to determine which subcohorts of patients will benefit from ICIs and guide clinical decision making.

Though a relatively young field, the role of immunotherapy in CCA is rapidly expanding. Novel approaches such as the utilization of conjugate antibodies and adoptive T-cell transfer are upcoming and are burgeoning to fill several gaps. A thorough understanding of the tumor microenvironment as well as heterogeneity in inter/intratumoral responses is important for optimizing responses to immunotherapy against this challenging malignancy.

## Figures and Tables

**Table 1:** Trials of immune checkpoint inhibitor (ICI) use as monotherapy in cases with biliary tract cancers (including cholangiocarcinoma) (references [17,19,20,21,22,23]).

Treatment	Trial Name	Target	Phase	Line of Therapy	ORR (%)	OS (m)	PFS (m)
Pembrolizumab	KEYNOTE-028	PD-1	I	second	13	5.7	1.8
Pembrolizumab	KEYNOTE-158	PD-1	II	second	5.8	7.4	2
Nivolumab	NCT02829918	PD-1	II	second	22	14.2	3.68
Nivolumab	JapicCTI-153098	PD-1	I	second	3	5.2	1.4
Durvalumab	NCT01938612	PDL1	I	second	4.8	8.1	2
Bintrafusp alfa	NCT02699514	PD-L1 TGFβ-RII	I	second	20	12.5	2.5

Abbreviations: PD-1, programmed cell death protein-1; TGF-R, transforming growth factor receptor; ICI, immune checkpoint inhibitor; ORR, objective response rate; OS, overall survival; PFS, progression-free survival.

**Table 2:** Trials of immune checkpoint inhibitor (ICI) use as combination therapy in cases with biliary tract cancers (including cholangiocarcinoma) (references [28,29]).

Treatment	Trial Name	Target	Phase	Line of Therapy	ORR (%)	OS (m)	PFS (m)
Nivolumab Ipilimumab	CA209-538	PD-1 CTLA4	II	second	23	5.7	2.9
Durvalumab + Tremelimumab	NCT01938612	PDL1, CTLA4	II	second	10.8	10.1	-

Abbreviations: PD-1, programmed cell death protein-1; ctla-4, cytotoxic T-cell lymphocyte-associated protein-4; ICI, immune checkpoint inhibitor; ORR, objective response rate; OS, overall survival; PFS, progression-free survival.

**Table 3:** Trials of immune checkpoint inhibitors (ICIs) with chemotherapy in cases with biliary tract cancers (including cholangiocarcinoma) (references [31,32,33,34,35,36,37]).

Treatment	Trial Name	Target	Phase	Line of Therapy	ORR (%)	OS (m)	PFS (m)
Nivolumab cisplatin/gemcitabine	JapicCTI-153098	PD-1	II	first	37	15.4	4.2
Nivolumab cisplatin/gemcitabine	NCT03311789	PD-1	II	first	55.6	8.5	6.1
Camrelizumab Gemcitabine/oxaliplatin	NCT03486678	PD-1	II	first	54	11.8	6.1
Camrelizumab Gemcitabine/oxaliplatin or FOLFOX	NCT03092895	PD-1	II	first	16.3	12.4	5.3
Durvalumab Cisplatin/gemcitabine	NCT03046862	PD-L1	II	first	73.3	18.1	11
Durvalumab + GEMCIS	TOPAZ-1	PD-L1	III	first	26.7 vs. 18.7	12.8 vs. 11.5	7.2 vs. 5.7

Abbreviations: PD-1, programmed cell death protein-1; ICI, immune checkpoint inhibitor; GEMCIS, gemcitabine–cisplatin; FOLFOX, 5-fluorouracil, leucovorin, and oxaliplatin; ORR, objective response rate; OS, overall survival; PFS, progression-free survival.

**Table 4:** Trials of immune checkpoint inhibitors (ICIs) with antiangiogenic agents in cases with biliary tract cancers (including cholangiocarcinoma) (references [41,42]).

Treatment	Trial Name	Target	Phase	Line of Therapy	ORR (%)	OS (m)	PFS (m)
Lenvatinib + Pembrolizumab	LEAP-005	PD-1	II	second	10	8.6	6.1
Toripalimab and lenvatinib + GEMOX	NCT03951597	PD-1	II	first	80	NA	10

Abbreviations: PD-1, programmed cell death protein-1; ICI, immune checkpoint inhibitor; GEMOX, gemcitabine–oxaliplatin; ORR, objective response rate; OS, overall survival; PFS, progression-free survival.

**Table 5:** Ongoing active clinical trials on immunotherapy as a first-line treatment for advanced biliary tract cancers.

Treatment	Trial Name	Phase	Primary Outcome
GEMCIS ± Pembrolizumab	NCT04003636	III	OS
GEMCIS ± Bintrafusp alfa	NCT04066491	II	OS
Pembrolizumab + GEMCIS	NCT03260712	II	PFS
Nivolumab + GEMCIS vs. Nivolumab + Ipilimumab	NCT03101566	II	PFS
Nivolumab + Gemcitabine + TS-1	NCT04172402	II	ORR

Abbreviations: GEMCIS, gemcitabine–cisplatin; OS, overall survival; PFS, progression-free survival; ORR, objective response rate.

## Data Availability

Not applicable.
